# Rate of Parental Consanguineous Marriage among Patients with Visual Impairments in Turkey

**Published:** 2016

**Authors:** Sezen AKKAYA

**Affiliations:** 1 Ophthalmology Department, Fatih Sultan Mehmet Training and Research Hospital, Istanbul, Turkey

**Keywords:** Parental Consanguineous Marriage, Visual Impairment, Turkey, Nystagmus

## Abstract

We aimed to describe the causes, characteristics, and rate of parental consanguineous marriage associated with patients with visual impairments in Turkey. This study involved 236 patients with visual impairments. The 10th revision of the International Classification of Diseases was used to categorize the causes of visual impairments (based on the main cause in both eyes). The mean age of the patients was 38.5 ± 24.2 years (range, 6–95 years), and most were in the 15–30-year age group (35.6%). There were more male patients (65%) than female patients. Blindness, severe visual impairment, and mild to moderate visual impairment were observed in 30 (12.7%), 84 (35.6%), and 122 (51.6%) patients, respectively. Choroidal and retinal diseases were identified as the main underlying cause of visual impairment (62.7%), followed by nystagmus (23.7%), optic tract and nerve diseases (11.0%), congenital cataracts (0.8%), and glaucoma (1.7%). Parental consanguinity was present for 26.3% of the patients, and it was significantly more common in the 15–30-year age group (50%) compared to the other age groups. In Turkey, the main cause of visual impairment was choroid and retinal diseases in all the age groups above 14 years, while nystagmus was the most common cause in the age group below 15 years. Parental consanguinity was significantly higher among the patients with macular dystrophy and those with retinitis pigmentosa than with retinopathy of prematurity, optic nerve diseases, age-related macular degeneration, and diabetic retinopathy. Genetic factors are known to be involved in the development of these diseases, indicating that the issue of consanguineous marriage remains a problem in Turkey.

## INTRODUCTION

The World Health Organization (WHO) defines a person with low vision as someone who, while being able to use his or her vision to plan and execute tasks, continues to experience a visual function impairment despite refractive correction or other treatment, in addition to having a visual acuity (VA) < 6/18 (20/60) to light perception or a visual field < 10° from the point of fixation in the better-seeing eye (1). Along with onchocerciasis, trachoma, cataracts, refractive error, and childhood blindness, low vision is a priority of the “VISION 2020: The Right to Sight” global initiative. In both developing and developed countries, blindness and low vision are significant health and socioeconomic concerns. The prevalence of low vision is significantly affected by demographic characteristics, cultural differences, and socioeconomic factors. The relevant demographic characteristics include age, as the underlying causes of visual impairment differ between age groups, with many cases being preventable (2-5). For these reasons, assessing the epidemiology of visual impairments and identifying their underlying causes is an important subject that is directly relevant to national healthcare planning (6-8).

To decrease dependency and improve quality of life among patients affected by visual impairments, special treatment programs that include clinical assessment, expert consultations, and rehabilitation are required (9-13). The prevalence of low vision in Turkey among patients aged ≥ 75 years was previously reported to be 10.3%, and among those aged ≥ 90 years, it was reported to be about 30% (14). We investigated the characteristics of patients with blindness or low vision in Turkey, such as their sex and age, as well as the underlying cause of their visual impairment and the presence or absence of parental consanguinity. It is expected that the results will be useful for health care planning, including the planning of rehabilitation services for people with visual impairments.

## Materials and Methods

This study involved consecutive patients who were fully blind or visually impaired and who were attending a vision rehabilitation clinic in Turkey in Istanbul to receive low vision aids (LVAs). Informed consent was obtained from all the participating patients. We also obtained a signed consent statement from each patient and authorization from the Fatih Sultan Mehmet Education and Training hospital’s ethics committee to use the patients’ personal and medical information in our study. After recruiting the participants, examinations were performed by a single ophthalmologist. An auto refractometer was used to measure refractive error, and a Snellen chart was used to assess the best-corrected visual acuity (BCVA) and uncorrected visual acuity (UCVA). Near vision was evaluated with an Amsler chart. Based on their BCVA, the patients were divided into three groups in line with the following WHO classification system: 1) blind (BCVA < 20/400); 2) severe low vision (20/200 < BCVA ≤ 20/400); and 3) mild to moderate low vision (20/60 < BCVA ≤ 20/200) (7).

The referring physicians were asked to provide details of the cause of visual impairment for each patient (based on their clinical and paraclinical assessments). The patients who did not have an exact diagnosis were referred to an ophthalmologist for additional diagnostic procedures. The patients were classified according to the primary cause of visual impairment for both eyes, using the 10th revision of the International Classification of Diseases (ICD-10) (8), which categorizes diseases according to specific code subheadings of general headings that classify disorders based on the anatomic site involved. The data are presented as means** ± **SDs and frequencies (with percentages). Pearson’s chi-square tests were used for the qualitative evaluation of the data, while Yates’ continuity correction and Fisher’s exact tests were used to compare the data. 0.05 was considered statistically significant. Number Cruncher Statistical System (NCSS) version XYZ and Power Analysis and Sample Size (PASS) version XYZ software programs (NCSS LLC, Kaysville, UT, USA) were used for all the statistical analyses.

## Results

In this study, 236 patients, comprising 156 male (66.1%) and 80 female patients (33.9%), were enrolled. All patients were of Turkish nationality. The age range was 6–95 years, and the mean age was 38.5 ± 24.2 years. The most common age group was the 15–30-year group (35.6%), followed by the > 50-year (28.8%) and 31–50-year (21.2%) groups. Parental consanguinity was present for 62 (26.3%) patients, and it was most common in the 15–30-year group (50.0%) and least common in the age group >50 years (0.001) (Table 1).

Varying degrees of visual impairments were observed among the patients, and a BCVA < 20/400 (blindness) was observed for 12.7% of the participants (Table 2). Parental consanguinity was significantly higher in the group with mild to moderate visual impairments (32.8%) compared to the other visual impairment groups (0.05).

The primary cause of visual impairment was identified for each age group. In all age groups except for the < 15 group, the most common cause was choroidal and retinal diseases (62.7%). As for the < 15 group, nystagmus was the most common cause. The rate of parental consanguineous marriage was evaluated for each cause of visual impairment (Table 3). The parental consanguinity rate was significantly higher for patients with retinitis pigmentosa (0.001) or Stargardt’s macular dystrophy (0.05) than with optic nerve diseases, retinopathy of prematurity, age-related macular degeneration (AMD) and diabetic retinopathy (DRP). The parental consanguinity rate was zero for patients’ with AMD or DRP.

**Table 1:** Number of patients with visual impairments by age group (rates among men and total patients associated with parental consanguineous marriage)

**Age group (year)**	**Number of patients (%)**		
	**Total**	**Men**	**Parental consanguineous marriage**
**< 15**	34(14.4%)	20(58.8%)	6(17.6%)
**15–30**	84 (35.6%)	60(71.4%)	42(50.0%)^a^
**31–50**	50 (21.2%)	30(60.0%)	10(20.0%)
**> 50**	68(28.8%)	46(67.6%)	4(5.9%)^a^
**Total**	236	156 (66.1%)	62(26.3%)

a P < 0.001

**Table 2:** Number of patients by severity of visual impairment (rates among patients associated with parental consanguineous marriage)

**Visual impairment (based on the WHO classification system)**	**BCVA range**	**Number of patients (%)**	
		**Total (n = 236)**	**Parental consanguineous marriage (n = 62)**
**Mild to moderate**	20/60 < BCVA ≤ 20/200	122 (51.6%)	40(32.8%)^a^
**Severe**	20/200 < BCVA ≤ 20/400	84 (35.6%)	16 (19.0%)
**Blindness**	BCVA < 20/400	30 (12.7%)	6(20.0%)

a P < 0.05; WHO: World Health Organization

**Table 3:** Rate of parental consanguineous marriage by cause of visual impairment

**Number of patients (%)**	**Nystagmus**	**Stargardt’s macular dystrophy**	**Optic tract and nerve diseases**	**ROP**	**Retinitis pigmentosa**	**AMD**	**DRP**	**Other**	**Total**
**Positive**	20(32.3%)	18(29%)	8(12.9%)	2(3.1%)	8(12.9%)	0	0	6	62
**Negative**	38(21.8%)	26(14.9%)	10(5.7%)	2(1.1%)	4(2.3%)	38	16	40	174
**Total**	58	44	18	4	12	38	16	46	236
**P value**		< 0.05^a^			< 0.001^a^	^ a^	^ a^		

AMD: Age-related macular degeneration; DRP: diabetic retinopathy; ROP: retinopathy of prematurity

a Statistically significant

## DISCUSSION

The results showed that, using the ICD-10 classification, overall, the leading causes of visual impairment among patients in Turkey are choroidal and retinal diseases followed by nystagmus. Other less common causes are congenital cataracts, optic tract and nerve diseases, and glaucoma. This pattern of the less common causes of visual impairment was observed in all age groups. However, the pattern of leading causes varied between the age groups. In the < 15-year age group, albinism and nystagmus were the most prevalent causes, in the 15–50 group, the most prevalent causes were Stargardt’s macular dystrophy and retinitis pigmentosa, while in the > 50-year age group, the most common causes were DRP and AMD.

A retrospective study of 4711 patients’ at a vision rehabilitation clinic in Germany showed that the main cause of visual impairment was AMD (40%). This was followed by tapetoretinal dystrophy, optic nerve atrophy, and DRP (15). A retrospective study of 573 patients at a vision rehabilitation clinic in Malaysia showed that the most common cause of visual impairment tended to vary depending on age. In the age group < 30 years, congenital disease was the most common cause, while among individuals between 30 and 60 years, the most common cause was retinitis pigmentosa, and for those aged > 60 years, the most common cause was AMD (16). A study in Tehran of 362 students from three schools for blind students showed that 8.9% had severe low vision, and retinal disease was the most common cause of low vision (51%) while other common causes were optic nerve atrophy, cataracts, glaucoma, corneal and anterior segment disease, globe malformations, and anophthalmia (17). In the present study, 41.8% of the participants were in the age group > 50 years, which might explain why DRP and AMD had high prevalence in our sample. This observation is in agreement with the worldwide visual impairment data for 2002 for DRP and AMD (3). Previous research has indicated that men are more predisposed to visual impairment than women, with Nguyen et al. (15) determining that 58.9% of those affected by visual impairment are men. The majority of the patients in the present study were also men (66.1%).

Parental consanguinity was present for 26.3% of the patients, and the percentage was highest in the 15–30 age group (50.0%) (0.001). The most common causes of visual impairment in this age group were Stargardt’s macular dystrophy and nystagmus, and genetics are known to play a role in these diseases. Parental consanguinity was lowest in the > 50-year age group (5.9%) (0.001). The two most common diseases in the > 50 group were AMD and DRP. Parental consanguinity was present for zero patients in both the AMD or DRP groups, as genetics do not play a major role in the development of these diseases. Overall, the most common causes of visual impairment were choroidal and retinal diseases. This observation may be explained as follows: 1) compared to other ophthalmic conditions, choroidal and retinal diseases are less curable; 2) due to parental consanguinity, choroidal and retinal diseases are more common in Turkey than in other countries; and 3) due to referral to vision rehabilitation clinics by ophthalmologists, patients with these kinds of ophthalmic problems may be more common in the sample used in this study.

The first explanation is most likely to be the most important. In many population-based studies in the world, a major cause of visual impairment is cataract (3). Other less common causes include (in order of decreasing importance) refractive errors, trachoma, glaucoma, macular degeneration, and retinal diseases (11). As the present study was performed among patients who had been referred to a vision rehabilitation clinic, two of these frequent diseases (i.e., refractive errors and cataracts) were not present among the leading causes of visual impairment in the study sample, since, in Turkey, patients with refractive errors and cataracts are treated rather than only being offered rehabilitation. Regarding the assumption in the second explanation that there is a higher prevalence of retinal and choroidal diseases in Turkey, this may also play a role in the leading cause of visual impairment identified in this study. There is a possibility that both congenital and acquired retinal diseases are very common in the Turkish population. Such a finding would concur with the observations from Brazil (18), Germany (15), India (19), Thailand (20), and the Netherlands (21), which indicate that retinal diseases are the most common cause of low vision. In contrast, studies conducted in Ethiopia (22) and Uganda (23) show that vision loss is more commonly associated with anterior segment diseases. This might be a reflection of the high frequency of vitamin A deficiency and infectious diseases in Ethiopia and Uganda.

In the present study, after retinal and choroidal diseases, optic nerve diseases were the second most common cause of visual loss. A study in Brazil of 3210 children with multiple disabilities showed that visual impairments were most commonly caused by optic nerve atrophy (37.7%), followed by cortical blindness (19.7%). The authors stressed the necessity of performing physical examination on other parts of the body in children with optic nerve atrophy (18), and we also recommend carrying out physical examinations of children with optic atrophy. At vision rehabilitation clinics and other health care facilities, the type of LVAs prescribed to patients with visual impairments is largely dependent on each patient’s visual function, rather than on the cause of the visual impairment. Thus, visual function should be assessed before prescribing LVAs (24-26). Previous research has shown that, for the majority of patients, near and distance BCVA improved significantly because of the use of LVAs, indicating that LVAs can be effective for improving visual function (27-29).

While younger patients still constitute a major proportion of patients attending vision rehabilitation clinics, we also believe that there is an increase in the number of elderly patients with visual impairment, as AMD is gradually becoming another major cause of visual impairment (14). According to previous research, the rate of consanguineous marriages in Istanbul is 25%, and in other cities in Turkey, it is 20–30%. Our results showed that, overall, parental consanguineous marriages were present for 26.3% of the patients, though the parental consanguinity rate was zero for patients with AMD or DRP. In Turkey, genetic diseases such as sickle cell anemia and thalassemia (and thalassemia trait, which does not usually cause health problems) are relatively common. Genetic diseases are a major problem in Turkey, as there are no cures for these diseases, they often cause psychological trauma, and the maintenance costs are high. Only 12.7% of the participants had BCVA < 20/400 (blindness) because fully blind patients are only rarely referred to vision rehabilitation clinics. It should be noted that since this study only evaluated patients who attended a rehabilitation clinic, the results are not generalizable to the entire population of patients with visual impairments in Turkey. Nevertheless, this study contributes to identifying the causes of incurable visual impairment in Turkey, and the results could be useful for planning screening programs and vision rehabilitation services. In conclusion, consanguineous marriage unfortunately continues to be a major issue in Turkey, and education about the risks of consanguineous marriage is essential. Furthermore, the results of these types of studies can be utilized for creating practical guidelines for vision rehabilitation clinics. Further studies involving larger populations are needed, particularly in other cities in Turkey.
